# The dopamine D_2_ receptor dimer and its interaction with homobivalent antagonists: homology modeling, docking and molecular dynamics

**DOI:** 10.1007/s00894-016-3065-2

**Published:** 2016-08-04

**Authors:** Agnieszka A. Kaczor, Manuela Jörg, Ben Capuano

**Affiliations:** 1 Department of Synthesis and Chemical Technology of Pharmaceutical Substances with Computer Modeling Laboratory, Faculty of Pharmacy with Division for Medical Analytics, Medical University of Lublin, 4A Chodźki St., 20059 Lublin, Poland; 2 School of Pharmacy, University of Eastern Finland, Yliopistonranta 1, PO Box 1627, 70211 Kuopio, Finland; 3 Medicinal Chemistry, Monash Institute of Pharmaceutical Sciences, Monash University, 381 Royal Parade, Parkville, Victoria 3052 Australia

**Keywords:** Bivalent ligands, Dopamine D_2_ receptor, GPCR, GPCR dimer, Homology modeling, Molecular docking, Molecular dynamics

## Abstract

In order to apply structure-based drug design techniques to G protein-coupled receptor complexes, it is essential to model their 3D structure and to identify regions that are suitable for selective drug binding. For this purpose, we have developed and tested a multi-component protocol to model the inactive conformation of the dopamine D_2_ receptor dimer, suitable for interaction with homobivalent antagonists. Our approach was based on protein–protein docking, applying the Rosetta software to obtain populations of dimers as present in membranes with all the main possible interfaces. Consensus scoring based on the values and frequencies of best interfaces regarding four scoring parameters, Rosetta interface score, interface area, free energy of binding and energy of hydrogen bond interactions indicated that the best scored dimer model possesses a TM4–TM5–TM7–TM1 interface, which is in agreement with experimental data. This model was used to study interactions of the previously published dopamine D_2_ receptor homobivalent antagonists based on clozapine,1,4-disubstituted aromatic piperidines/piperazines and arylamidoalkyl substituted phenylpiperazine pharmacophores. It was found that the homobivalent antagonists stabilize the receptor-inactive conformation by maintaining the ionic lock interaction, and change the dimer interface by disrupting a set of hydrogen bonds and maintaining water- and ligand-mediated hydrogen bonds in the extracellular and intracellular part of the interface.

**Electronic supplementary material:**

The online version of this article (doi:10.1007/s00894-016-3065-2) contains supplementary material, which is available to authorized users.

**Graphical Abstract:**
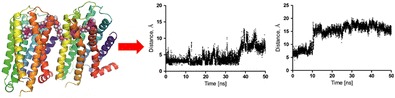
Structure of the final model of the dopamine D2 receptor homodimer, indicating the distancebetween Tyr37 and Tyr 5.42 in the apo form (*left*) and in the complex with the ligand (*right*).

## Introduction

Dopamine receptors can be categorized into five different subtypes (namely D_1_ to D_5_), which are all members of the G protein-coupled receptor (GPCR) superfamily. These receptors can be further classified into D_1_-like (D_1_ and D_5_) and D_2_-like (D_2_, D_3_ and D_4_) receptors depending on activation or inhibition of the second messenger cyclic adenosine monophosphate (cAMP), respectively [1, 2]. Dopamine receptors are implicated in many disease states, including Parkinson’s disease, restless legs syndrome, sexual dysfunction, dementia, depression, bipolar disorder, Huntington’s disease and schizophrenia. Consequently, drug discovery has been focusing on targeting the dopaminergic system for over half a century, resulting in a range of dopaminergic pro-drugs, agonists, antagonists, and enzyme inhibitors presently on the market [3].

Traditionally, research in academia and industry has been focusing on the discovery and development of drugs targeting the orthosteric pocket of a receptor monomer. The function of GPCRs is classically described by the ternary complex model as the interplay of three basic components: a receptor monomer, an agonist and a G protein. According to this model, receptor activation results from an interaction with an agonist, which translates into the activation of a particular G protein in the intracellular compartment that, in turn, is able to initiate particular signaling cascades. The growing body of experimental evidence on GPCR functioning has revealed that GPCR signaling can be modulated in ways much more complicated than those contemplated in the ternary complex model, which has led to a paradigm shift in GPCR oriented drug discovery [4]. In particular, the accumulation of experimental and computational evidence from cross-linking experiments, BRET and FRET studies [5] and coarse-grained molecular dynamics simulations reporting negative and positive cooperativity, has suggested the possibility that GPCRs may be capable of oligomerization [6, 7]. In recent years, both homo- and heterodimerization have been described for an increasing number of GPCRs, and, in some cases, these associations have been related to particular functional outcomes [8, 9]. For this reason, GPCR oligomers have also been reported as potential drug targets, which, due to their restricted tissue distribution, could provide a new source of drug specificity. Despite the increasing amount of functional interactions that have been described between dimers, the development of drugs with the ability to target receptor oligomers is still very challenging. Therefore, a deeper characterization of the basis of receptor dimerization and of its impact on signaling, together with the development of original treatment strategies, will be necessary for the pharmacological exploitation of this phenomenon [10].

As a consequence of the paradigm shift in GPCR-oriented drug discovery, the scientific community has gained interest in the development of novel types of molecules including homo- and heterobivalent ligands. These modern concepts result in new challenges for molecular modelers to provide medicinal chemists with appropriate receptor models of homo- and hetero-dimers and higher oligomers, which allow accurate predictions of possible binding modes of these novel types of molecules.

Molecular modeling approaches used to study GPCR oligomerization can be categorized into sequence-based and structure-based methods [11]. The first group is based on the GPCR sequence analysis performed in order to detect evolutionary changes in GPCR interfaces. However, to use structure-based drug design approaches for GPCR dimers and oligomers, it is necessary to model their 3D structure. Structure-based approaches involve protein–protein docking and molecular dynamics techniques as well as electrostatics analysis with adaptive Poisson-Boltzmann solver (APBS) and normal mode analysis (NMA). Previously, we elaborated a multi-component protocol [12] for modeling GPCR dimers that is based on protein–protein docking with Rosetta software [13]. In this protocol, populations of dimers compatible with membrane integration are generated, taking into account all possible interfaces. The method involves external scoring (as protein–protein docking itself fails in case of GPCR dimers [14]), followed by consensus scoring of the resulting dimers according to (1) Rosetta total score, (2) Rosetta interface score, (3) surface of the dimer interface, (4) polar contribution to the dimer interface, (5) fractal dimension of the dimer interface [15, 16], (6) evolutionary conservation score [17, 18], (7) shape complementarity, (8) electrostatic complementarity, (9) potential energy [19], (10) free energy of binding [19], and (11) energy of hydrogen bond interactions [12, 20]. The protocol was tested by prediction of the dimer structure of GPCR dimers with existing X-ray structures, and it was concluded from these studies that the Rosetta interface score, interface area, free energy of binding and the energy of hydrogen bond interactions were the best performing scoring factors [12].

**Fig. 1:**
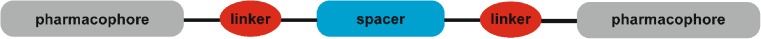
Schematic representation of a homobivalent ligand

Dopamine D_2_ receptor homodimers might be particularly important for the pathomechanism of Parkinson’s disease and schizophrenia, and may serve as attractive targets for antiparkinsonian drugs and antipsychotics. In our previous study based on experimental data, we constructed a D_2_ receptor dimer model suitable for interaction with agonists, and studied its interactions with homobivalent ropinirole-based agonists [21]. The current study was focused on the molecular modeling of a dopamine D_2_ receptor homodimer suitable for interactions with antagonists using our protocol, and investigating the receptor’s interactions with published examples of homobivalent antagonists. Homobivalent ligands are molecules containing two entities of the same pharmacophore separated by a spacer unit (Fig. 1). Homobivalent ligands are interesting pharmacological tools for the probing of homodimers; however, their use as drugs is potentially limited due to their high molecular mass, lipohilicity and polar surface area. The work presented includes the previously published dopamine D_2_ receptor homobivalent antagonists based on clozapine [22], 1,4-disubstituted aromatic piperidines/piperazines [23], and arylamidoalkyl substituted phenylpiperazine [24] pharmacophores. We studied interactions of homobivalent antagonists with a dopamine D_2_ receptor homodimer using molecular docking and molecular dynamics.

## Computational methods

### Homology modeling

The homology model of the human dopamine D_2_ receptor (P14416) in the inactive conformation and in complex with the antagonist eticlopride were constructed using homology modeling with Modeller 9.10 [25] as described previously [26, 27]. The X-ray structure of the dopamine D_3_ receptor in complex with the antagonist eticlopride (PDB ID: 3PBL) [28] was used as a template. Multiple sequence alignment of 50 rhodopsin-like GPCRs was performed with the GPCR module of MOE Molecular environment [29]. Manual refinement was used in particular to satisfy the disulfide bridges, which were not found automatically by the software. The dopamine D_2_ receptor model was constructed without the N-terminus (the first 36 residues were omitted, the model starts with Tyr37), and without intracellular loop 3 (ICL3, residues Arg217–Lys362 were pmitted). A total of 100 homology models of the D_2_ receptor in complex with eticlopride were generated, and subsequently assessed by the Modeller objective function and Discrete Optimized Protein Energy profiles [30]. The best model was subjected to quality assessments using the MOE Molecular Environment module for Ramachandran plots [29].

### Initial set of dimers

We constructed an initial set of 144 plausible dimer interfaces by rotating one monomer around the other. This task was performed by creating a tcl script for VMD [31] as previously described [12]. The resulting set of dimers was compatible with membrane integration as the starting dimer was membrane-compatible and only rotation around* z*-axis was allowed.

### Protein–protein docking with Rosetta

Protein–protein docking with Rosetta [13] was used to obtain 10 dimers with each interface, resulting in 1440 final dimers. In order to obtain dimers compatible with membrane integration (as in input dimers were generated as above) protein–protein docking was performed in ‘refine only’ mode. Otherwise, default parameters were used.

### Details of scoring parameters

As previously suggested, we used four scoring parameters to assess the obtained set of dimers: Rosetta interface score, interface area, free energy of binding, and energy of hydrogen bond interactions [12]. The Rosetta interface score is one of the recorded parameters after protein–protein docking with Rosetta [13]. The interface area was calculated using VMD [31], being the difference of the sum of the molecular surfaces of two monomeric TM domains and the molecular surface of the TM domains in the dimer, as previously reported [12]. The free energy of binding, and the energy of hydrogen bond interactions (fraction of free energy of binding caused by hydrogen bonds), were calculated with Rosetta interface analyzer [13] as previously described [12]. Two methods of consensus scoring were used: (1) average scores of the 100 best scored dimers with respect to each interface, and (2) frequencies of interfaces among the 100 best scored dimers. Before consensus scoring was performed, the parameters were normalized to adopt values from 0 to 1.

### Minimization of the final dimer model

Before using for molecular docking of bivalent ligands the final homodimer model was minimized in order to adjust side chain conformations of the residues forming the interface using MOE Molecular environment [29]. Only side chains of the interface residues were subjected to minimization.

### Compound preparation

The investigated compounds (reference ligands chlorprothixene and olanzapine and compounds **1a-1f, 2a-2 g, 3a-3 g, 4a-4i** and **5a-5n**) were modelled using the LigPrep protocol from Schrödinger Suite [32]. The Epik module was used for different protonation states of the ligands at physiological pH. [33]. The compounds were further optimized with the Wavefunction Spartan10 software [34]. The procedure involved geometry optimization performed with B3LYP DFT using the 6-31G(d,p) basis set.

### Molecular docking

First, the complexes of the dopamine D_2_ receptor with chlorprothixene or olanzapine were constructed with Glide/induced-fit docking [35, 36] using the software Schrödinger suite. The grid was generated with default settings. The initial position of bivalent ligands was found manually and refined using Glide.

### Molecular dynamics

Molecular dynamics studies of ligand–receptor complexes were performed using Desmond v. 3.0.3.1 [37] and OPLS force field as described previously [38, 39]. The complexes were inserted into the POPC (1-palmitoyl-2-oleoyl-sn-glycero-3-phosphocholine) membrane, hydrated, and ions were added to neutralize protein charges and then to the reaching of a concentration of 0.15 M NaCl. The size of the simulation boxes was approximately 85 Å × 92 Å × 128 Å. The complexes were minimized and subjected to MD first in the NVT ensemble (constant number of particles, volume, and temperature) ensemble for 1 ns and then in NPT ensemble (constant number of particles, pressure, and temperature) for 20 ns with the restrictions on the protein backbone in each case [40]. The production run was performed in NPT ensemble with no restrictions for 50 ns. Analysis of molecular dynamics simulations was performed with the software tools from Schrödinger suite.

## Results and discussion

### Homology modeling

The obtained homology model of the D_2_ receptor in the inactive conformation has been described in detail previously [26, 27]. The sequence identity between the template and the target was 79 %, and the sequence similarity was 90 %. The model possesses two disulfide bridges, one between Cys107 and Cys182, and the other between Cys399 and Cys401 [26, 27].

### Modeling D_2_ receptor homodimer in the inactive conformation

We demonstrated previously that protein–protein docking methodology should be used with great care for modeling dimers and oligomers of GPCRs and, probably, the successful building of such complexes would require the support of experimental or sequence-based data about the residues involved in the interface [14]. We showed that the docking success is promoted by high symmetry order of the complex whereas other parameters, such as the hydrophobic/hydrophilic character of the interface turned out to have no correlation with the success of protein–protein docking [14]. In order to enhance the protein–protein docking performance for modeling GPCR dimers, we elaborated a protocol based on protein–protein docking with Rosetta supported by external scoring [12]. We applied this protocol to model D_2_ receptor dimers in the inactive conformation. A population of 1440 dimers with all possible interfaces was scored according to Rosetta interface score, interface area, free energy of binding, and hydrogen bond energy. Two methods of scoring were used, based on parameter values and best models frequency (see Computational methods). The scoring based on normalized parameter values and on best model frequency is presented in Fig. S1 and Fig. S2 in the Supplementary Information, respectively. In addition, Table 1 gives a summary of the best scored interfaces according to both approaches.

**Table 1:** Summary of best scored interfaces for the dopamine D_2_ receptor homodimer model

Method of scoring	Scoring parameter (Figure)	Best scored interfaces
Normalized parameter values	Rosetta interface score (Fig. S1a)	(1) TM3-TM4-TM7-TM1 (2) TM2-TM3-TM2-TM3 (3) TM4-TM5-TM7-TM1
	Interface area (Fig. S1b)	(1) TM2-TM3-TM2-TM3 (2) TM4-TM5-TM4-TM5 (3) TM4-TM5-TM7-TM1
	Free energy of binding (Fig. S1c)	(1) TM2-TM3-TM2-TM3 (2) TM3-TM4-TM7-TM1 (3) TM5-TM6-TM5-TM6
	Hydrogen bond energy (Fig. S1d)	(1) TM3-TM4-TM3-TM4 (2) TM5-TM6-TM5-TM6 (3) TM4-TM5-TM5-TM6
	Consensus scoring (Fig. 2a)	(1) TM2-TM3-TM2-TM3 (2) TM4-TM5-TM7-TM1 (3) TM4-TM5-TM4-TM5
Best model frequencies	Rosetta interface score (Fig. S2a)	(1) TM4-TM5-TM7-TM1 (2) TM3-TM4-TM5-TM6 (3) TM3-TM4-TM4-TM5
	Interface area (Fig. S2b)	(1) TM4-TM5-TM7-TM1 (2) TM3-TM4-TM4-TM5 (3) TM4-TM5-TM6-TM7
	Free energy of binding (Fig. S2c)	(1) TM4-TM5-TM7-TM1 (2) TM3-TM4-TM7-TM1 (3) TM3-TM4-TM5-TM6
	Hydrogen bond energy (Fig. S2d)	(1) TM4-TM5-TM5-TM6 (2) TM3-TM4-TM4-TM5 (3) TM3-TM4-TM7-TM1
	Consensus scoring (Fig. 2b)	(1) TM4-TM5-TM7-TM1 (2) TM3-TM4-TM4-TM5 (3) TM3-TM4-TM7-TM1

**Fig. 2:**
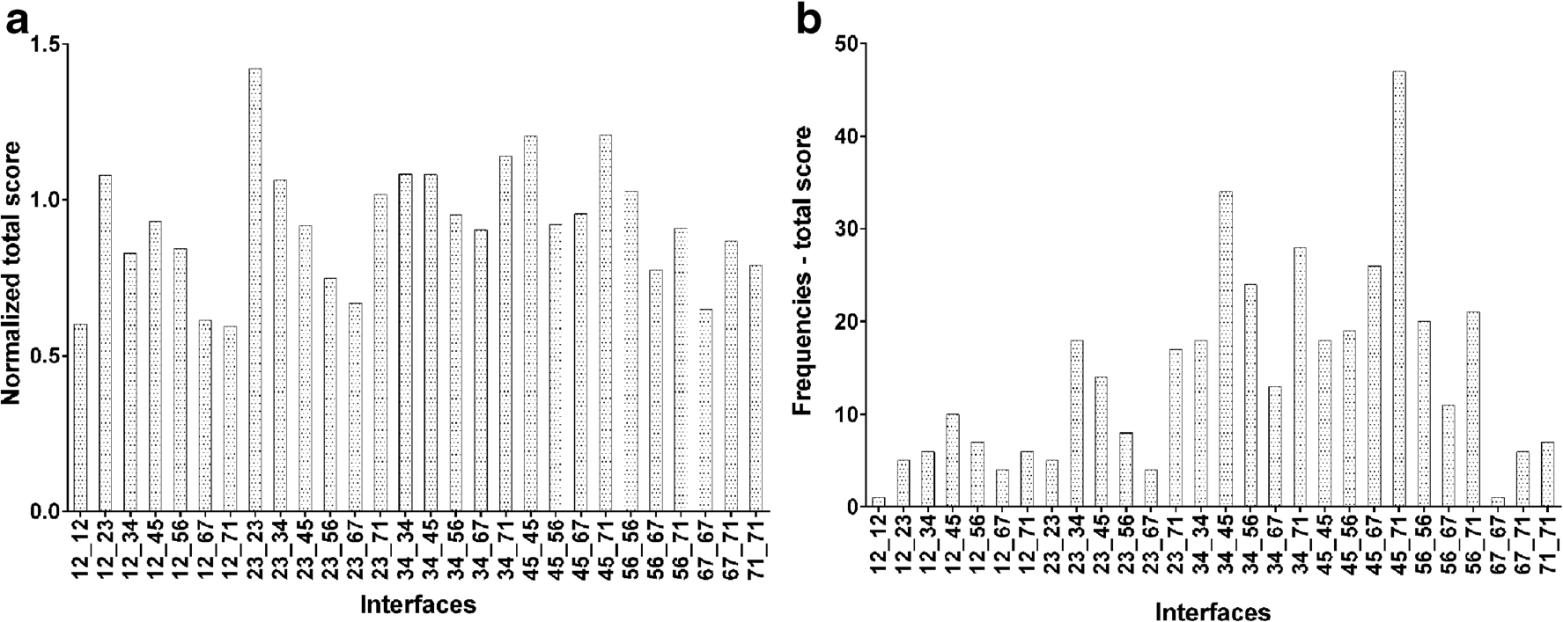
**a** Consensus scoring of normalized values, and **b** frequencies of best interfaces. Scoring was performed for 28 interfaces (*x*-axis) labeled according to transmembrane (TM) helices forming the interface, e.g., 12_12 for the interface formed by TM1 and TM2 from one monomer, and TM1 and TM2 from the other monomer

The consensus scoring for both parameter values and frequencies is shown in Fig. 2 and summarized in Table 1. The TM2–TM3–TM2–TM3 interface was disregarded as it has never been reported for any GPCR as TM2 and TM3 helices are involved in orthosteric ligand binding and/or allosteric modulation by sodium ions [41]. Thus, the dimer model with the interface formed by transmembrane helices TM4–TM5–TM7–TM1 was selected for further studies. This result is supported by high scoring results of both TM4–TM5 and TM7–TM1 interfaces partnered with other interfaces (see Fig. S1–S2 and Fig. 2) as well as by recent experimental data for a close homolog of the D_2_ receptor, namely the D_3_ receptor dimer [42]. Marsango et al. [42] used molecular modeling, mutagenesis and analysis of inactive state receptor crystal structures to indicate that D_3_ monomers can interact with each other via at least two distinct interfaces: the first comprising residues from transmembrane domains TM1 and TM2 along with those from TM7, and a second involving transmembrane domains TM4 and TM5. Moreover, Guitart et al. [43] also reported that the D_1_ receptor TM5 peptide was also able to reduce D_1_–D_1_ receptor complementation. This implies, according to the authors, [43] that D_1_ receptor TM5 forms part of the D_1_–D_3_ receptor heteromer, and D_1_–D_1_ receptor homomer interfaces, which can be also extended to the D_2_–D_2_ receptor homdimer.

### Structure of the dopamine D_2_ receptor homodimer

**Fig. 3:**
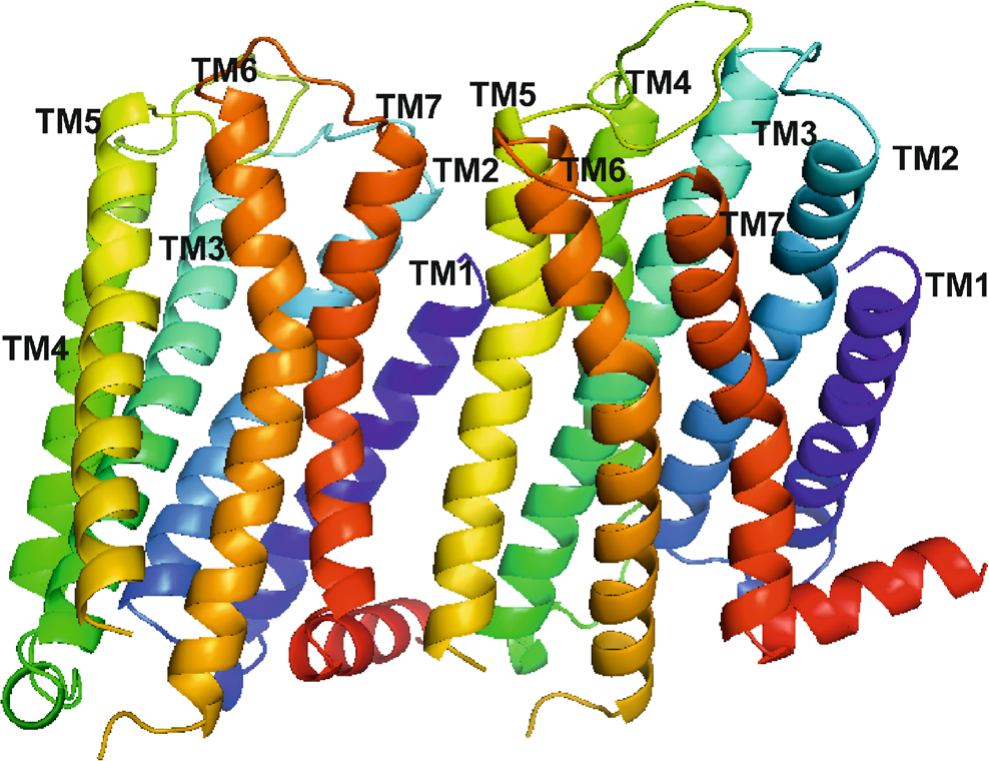
Structure of the final model of the dopamine D_2_ receptor homodimer. Transmembrane helices (*TMs*) are shown in* ribbon* representation, colored in spectrum mode and labeled with their respective* numbers*

The structure of the final dopamine D_2_ receptor dimer is presented in Fig. 3. The interface is stabilized by the hydrogen bond between the main chain of Tyr37 (N terminus) and the side chain of Tyr 5.42 (Ballesteros-Weinstein nomenclature [44]) from the other subunit. The following residues form the interface (as indicated by PISA online tool [45]): from subunit A: Leu 3.41, Tyr 3.51, Ala 3.55, Met138 (ICL1), Pro139 (ICL1), Met140 (ICL1), Leu141 (ICL1), Ile 4.48, Leu 4.52, Ala 5.38, Val 5.41, Tyr 5.42, Ile 5.45, Val 5.46, Val 5.49, Ile 5.52, Thr 5.54, Leu 5.56, Ile 5.59, Lys 5.60, Ile 5.63, Val 5.64; and from subunit B: Tyr37 (N terminus), Ala38 (N terminus), Thr39 (N terminus), Leu 1.38, Leu 1.41, Phe 1.48, Val 7.32, Leu 7.33, Ala 7.36, Trp 7.39, Leu 7.40, Val 7.43, Val 7.48, Ile 7.51, Thr 7.54, Thr 7.55, Arg 8.51, Leu 8.55, Leu 8.58, His442 (C terminus), and Cys443 (C terminus). This is in agreement with experimental data as it has been indicated that ICL3 from one subunit and the C terminus from the other subunit participate in the dopamine dimer formation, and are involved in electrostatic interactions [46]. Although the model was constructed without ICL3, it indicates the involvement of the C terminus in the homodimer formation.

The constructed model represents one possibility of a D_2_ receptor homodimer structure; another model of the active state with a TM4–TM5–TM4–TM5 interface was proposed earlier by us [21] and Durdagi et al. [47] for both the active and inactive state. These two models differ in the distance between two orthosteric sites and the orthosteric to the allosteric sites, which are slightly greater in the model with the TM4–TM5–TM7–TM1 interface (Table 2). The hypothesis about the possible existence of multiple GPCR interfaces has been supported recently by the demonstration that the dimer life-time is even shorter than previously thought [48]. Single-molecule imaging made it possible to determine the 2D monomer-dimer equilibrium constant, the dimer dissociation rate constant (typically ∼ 10 s^−1^), and the formation rate constant, which has demonstrated that GPCRs exist in dynamic equilibria between monomers and dimers [48]. Within 1 s, GPCRs typically undergo several cycles of monomer and homodimer formation with different interfaces, supporting the hypothesis of multiple oligomerization interfaces [48]. Thus, both models, with TM4–TM5–TM4–TM5 and TM4–TM5–TM7–TM1 represent two equally probable dopamine D_2_ receptor homodimer assemblies. Obviously, the interface does not depend on the dimer activation state and, in principle, both kinds of models can be used for molecular docking of bivalent agonists and antagonists on condition that they are built using receptor monomer models in the active or inactive conformation. Thus, in order to dock bivalent agonists to the dimer model with the TM4–TM5–TM7–TM1 interface, the model has to be reconstructed using receptor monomer models in the active conformation. The same applies to our previously constructed dimer model with the TM4–TM5–TM4–TM5 interface: in order to dock bivalent antagonists to this model, the model has to be rebuilt by applying receptor monomer models in the inactive conformation.

**Table 2:** Distances between different binding sites in the dopamine D_2_ receptor homodimer model (in Ångstroms)

Distance	D_2_ inactive	D_2_ active [21]
Orthosteric-orthosteric in different protomers through the membrane region	~38	~34
Orthosteric-orthosteric in different protomers without crossing a membrane region	~60-65	~50-60
Orthosteric-allosteric in one protomer	~18	~18
Orthosteric-allosteric in different protomers across the homodimer	~40-45	~30-40

### Molecular docking of the reference ligands

**Fig. 4:**
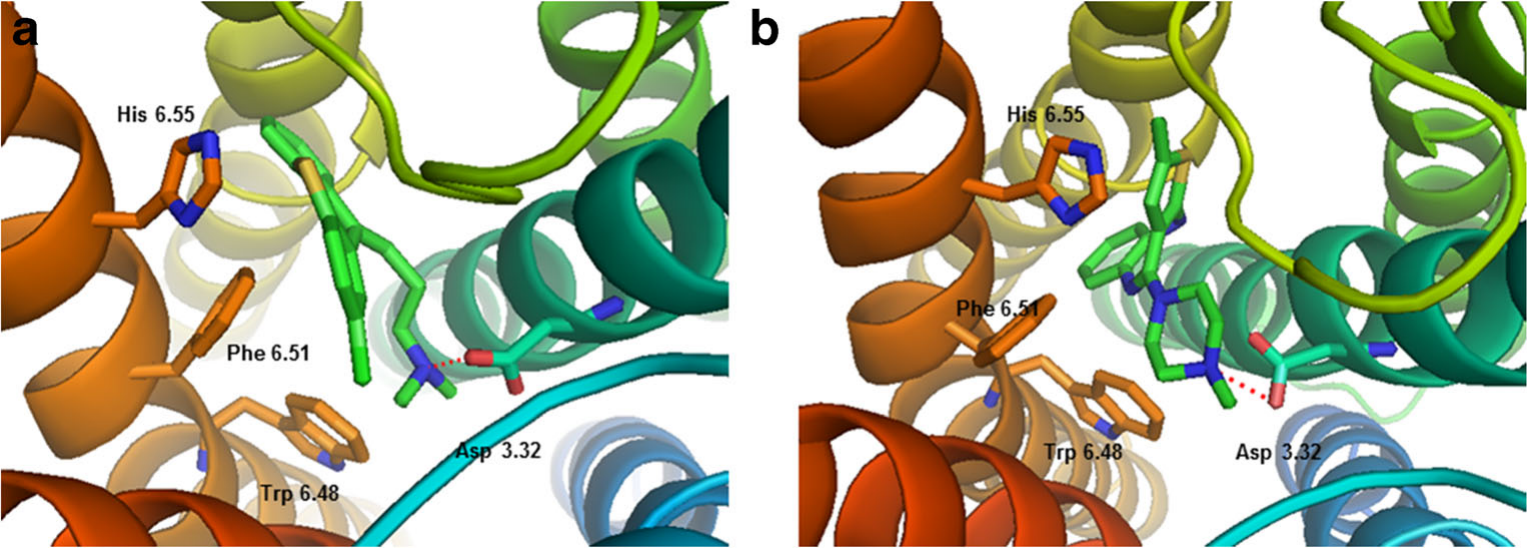
Reference ligands chlorprothixene (**a**) and olanzapine (**b**) docked into the orthosteric site of the dopamine D_2_ receptor homology model. Key interactions of the protonatable nitrogen atom of the ligands with the conserved Asp 3.32 (presented as* sticks*) are shown as* red dashed lines*. Other important residues, Trp 6.48, Phe 6.51 and His 6.55 are also shown as* sticks*. TMs are colored in spectrum mode. Hydrogen atoms are not shown for clarity

Two reference ligands, chlorprothixene and olanzapine, were docked to the model of the dopamine D_2_ receptor homodimer receptor using the SP (standard precision) protocol of Glide from the Schrödinger suite software. The docking poses were refined using the induced-fit docking approach in the Schrödinger suite. The final docking poses were identified by visual inspection among the poses, where the protonatable nitrogen atom of the ligands interacted with the conserved Asp 3.32 of the receptors. The docking poses of chlorprothixene and olanzapine are shown in Fig. 4. It can be seen that a key interaction for both ligands is an electrostatic interaction between the protonatable nitrogen atom of the ligand and Asp 3.32. Moreover, Trp 6.48, Phe 6.61 and His 6.55 were also found to be important for the binding of both ligands.

### The studied bivalent ligands

**Fig. 5:**
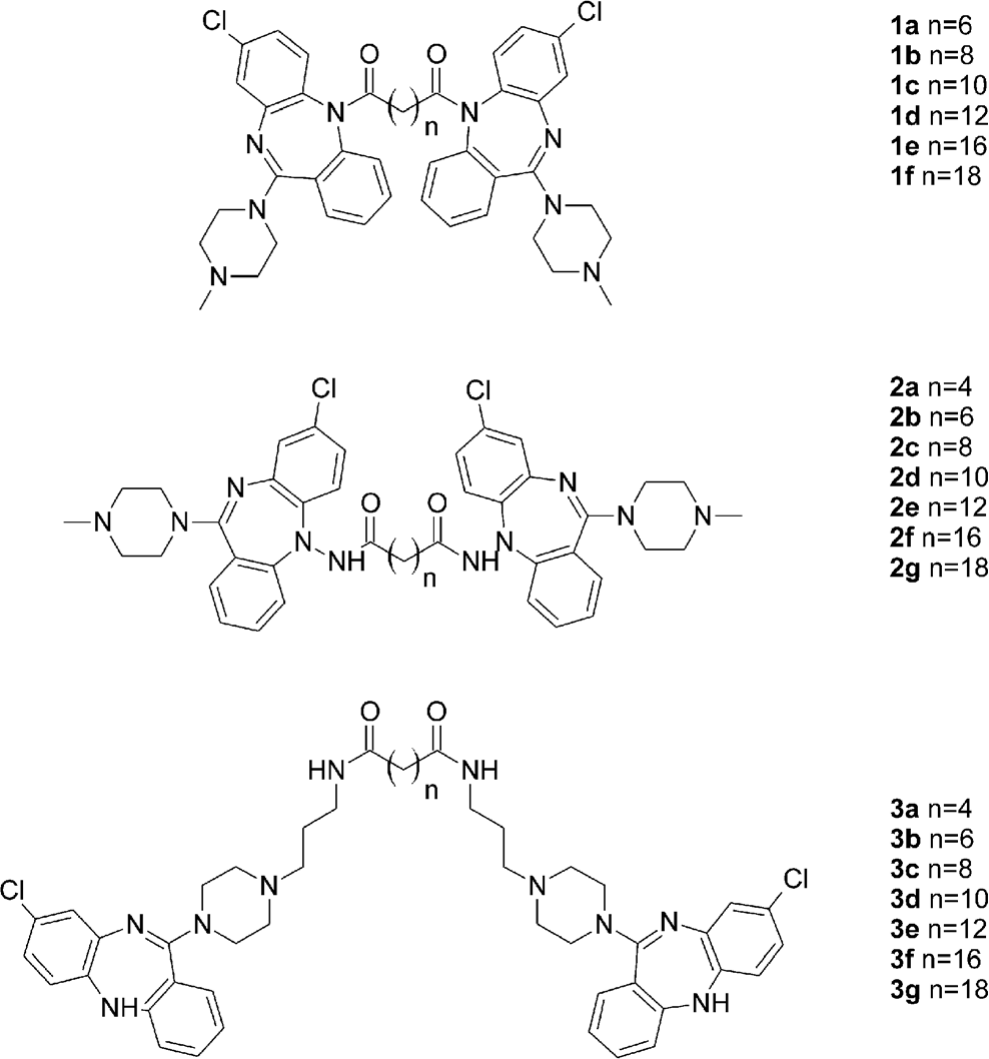
Clozapine-based homobivalent ligands **1a**–**1f**, **2a**–**2 g** and **3a**–**3 g** [22]

**Fig. 6:**
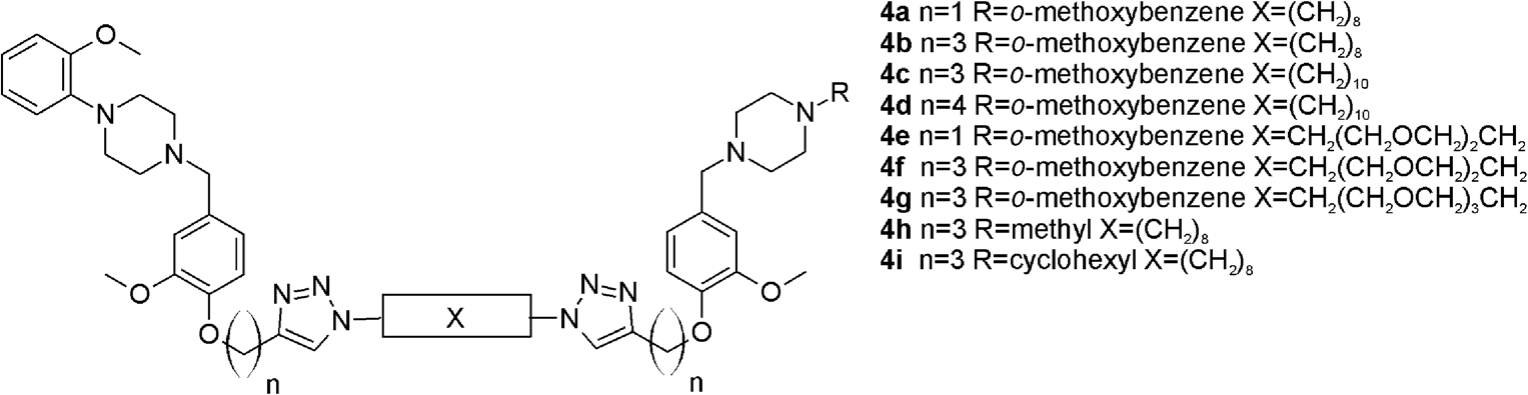
1,4-Disubstituted aromatic piperidine/piperazines-based homobivalent ligands **4a–4i** [23]

**Fig. 7:**
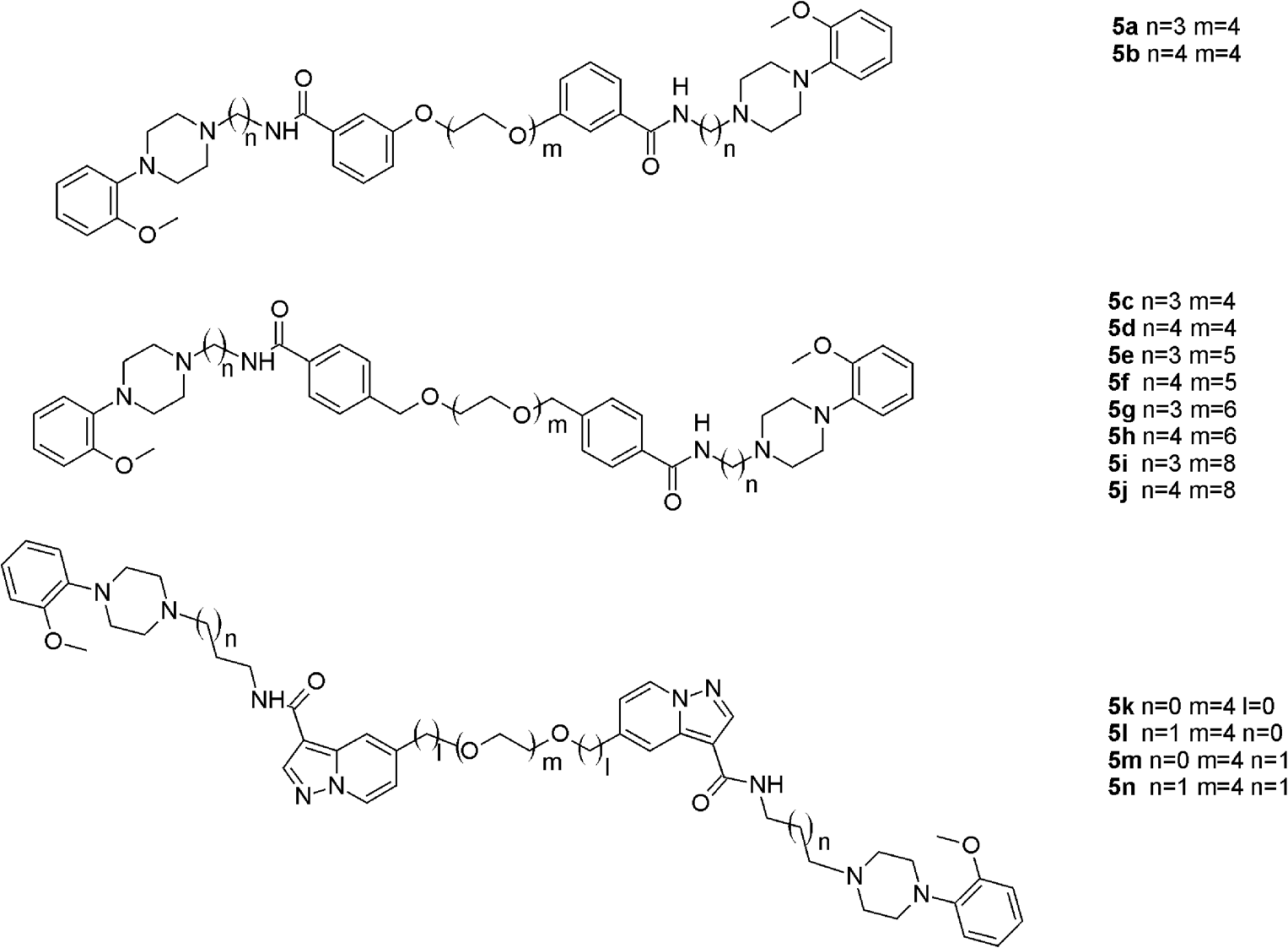
Arylamidoalkyl substituted phenylpiperazines-based homobivalent ligands **5a–5n** [24]

The studied bivalent ligands are based on clozapine [22], 1,4-disubstituted aromatic piperidines/piperazines [23], and arylamidoalkyl substituted phenylpiperazine [24] pharmacophores, which are presented in Figs. 5, 6 and 7. The reported biological activities of these ligands are given in Table 3.

**Table 3:** Biological activity of the investigated compounds towards D_2_ dopamine receptor.* NA* Not available,* NAP* not applicable

Compound number	Spacer length	Affinity, *K*_i_ (nM)	Potency IC_50_ (nM)	Reference
1a	8	NA	2662	[22]
1b	10	NA	>10,000	[22]
1c	12	NA	>10,000	[22]
1d	14	NA	>10,000	[22]
1e	18	NA	>10,000	[22]
1f	20	NA	>10,000	[22]
2a	6	NA	2078	[22]
2b	8	NA	2440	[22]
2c	10	NA	>10,000	[22]
2d	12	NA	>10,000	[22]
2e	14	NA	>10,000	[22]
2f	18	NA	>10,000	[22]
2 g	20	NA	>10,000	[22]
3a	14	3.6	87	[22]
3b	16	1.41	23	[22]
3c	18	1.35	44	[22]
3d	20	NA	1119	[22]
3e	22	NA	11,000	[22]
3f	26	269	7800	[22]
3 g	28	NA	>10,000	[22]
4a	18	25	NA	[23]
4b	22	41	NA	[23]
4c	24	17	NA	[23]
4d	26	8.2	NA	[23]
4e	18	3.4	NA	[23]
4f	22	3.7	NA	[23]
4 g	25	6.2	NA	[23]
4 h	22	17	NA	[23]
4i	22	1.1	NA	[23]
5a	NAP	6.4	NA	[24]
5b	NAP	1.8	NA	[24]
5c	NAP	19	NA	[24]
5d	NAP	11	NA	[24]
5e	NAP	32	NA	[24]
5f	NAP	15	NA	[24]
5 g	NAP	20	NA	[24]
5 h	NAP	25	NA	[24]
5i	NAP	66	NA	[24]
5j	NAP	16	NA	[24]
5 k	NAP	2.7	NA	[24]
5 l	NAP	0.59	NA	[24]
5 m	NAP	3.9	NA	[24]
5n	NAP	4.6	NA	[24]

### Molecular docking of bivalent ligands

Bivalent ligands **1a**–**1f**, **2a**–**2g**, **3a**–**3g**, **4a**–**4i** and **5a**–**5n** were docked into the dopamine D_2_ receptor homodimer model. None of the molecular docking software used (Surflex [49], Molegro [50], Glide [36]) was able to identify a correct docking pose of the bivalent ligand automatically, thus the initial ligand position was found manually and refined with Glide. In all cases, the docking poses selected included electrostatic interaction of the protonated nitrogen atom(s) of the ligand with Asp 3.32 from at least one monomer.

**Fig. 8:**
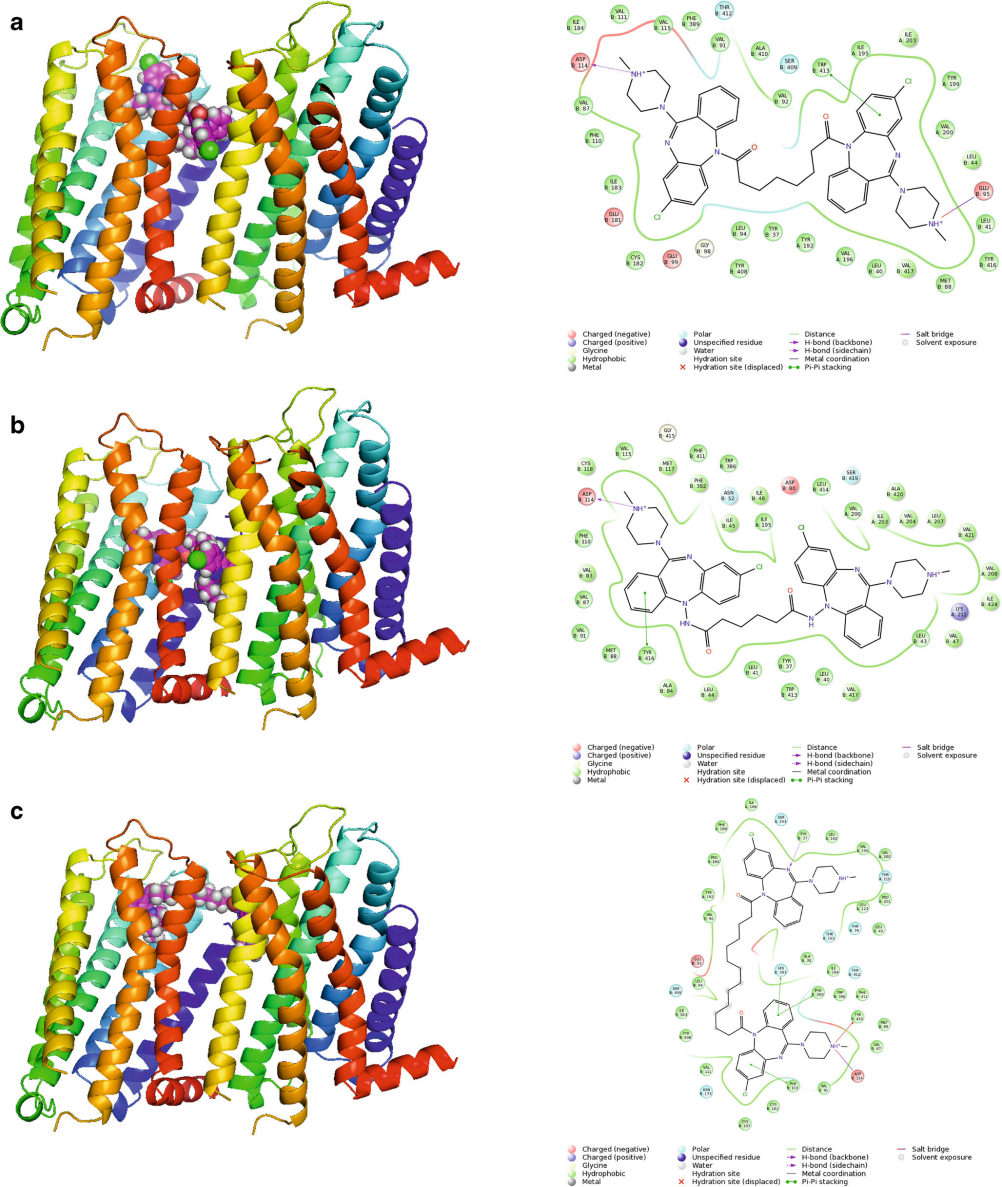
**a**, **b** Docking poses for the smallest ligands **1a** (**a**) and **2a** (**b**), and **c** a medium size ligand **1d** (the compound directs toward the allosteric site in the second monomer). Protein shown in* ribbon* representation, colored in spectrum mode. Ligands shown as* spheres* with* magenta carbon atoms*. 2D interaction maps are also presented

The first group of studied compounds involves homobivalent ligands based on clozapine **1a**–**1f**, **2a**–**2 g** and **3a**–**3 g** [22]. These ligand groups differ in their linker attachment point. Compounds **1a**–**1f** and **2a**–**2 g** have the N5 nitrogen atom as the linker attachment point as it has been suggested that clozapine modifications at this position may reduce agranulocytosis, which is a well-known side effect of clozapine [22]. Compounds **1a**–**1f** are directly acylated at the N5 nitrogen atom, whereas compounds **2a**–**2g** bear a hydrazine moiety at this position. Compounds **3a**–**3g** have the linker attached at the distant N4′-piperazine nitrogen atom. Molecular docking indicated that marginal activity of compounds **1a**–**1f** and **2a**–**2g** in comparison to clozapine may be caused by unfavorable conformation of these compounds caused by the linker attachment point, which makes it difficult for the ligand to interact with the conserved Asp 3.32, and to adopt well to the orthosteric pocket. Indeed, only very few poses fulfilling the requirement of this interaction were obtained for compounds **1a**–**1f** and **2a**–**2g**. This is in line with reports that only certain substituents at the N5 position of clozapine are tolerated [22, 51]. However Su et al. [52] reported that the N5-clozapine derivative with a relatively large substituent (*o*-TolSO_2_) displayed dopamine D_2_ receptor affinity of 63 nM, whereas the affinity of clozapine to this receptor is 208 nM. Thus, this effect is the least important for the smallest compounds **1a**, **2a** and **2b** (linker length 6–8 atoms) that can adopt a conformation able to interact within one monomer in the homodimer. Examples of docking poses for compounds **1a** and **2a** are shown in Fig. 8. It can be seen that both ligands bind in the orthosteric site of one monomer and are large enough to direct to another monomer, but are not able to interact with either the allosteric or the orthosteric site of the other monomer. Instead, ligand **1a** interacts with the allosteric site within one monomer so such compounds might behave as bitopic ligands. Ligands of medium size like **1d** (linker length of 14 atoms) may direct towards the allosteric site of the other monomer (Fig. 8c). Their lack of potency can be attributed to the hampered interaction with Asp 3.32, and a molecular shape that does not fit the receptor orthosteric pocket. In contrast, compounds **3a**–**3g** all displayed better potency than clozapine and spacer-dependent inhibitory activity [22]. The best potency and dopamine D_2_ receptor affinity was observed for compounds **3b** and **3c**, with 16- and 18-atom spacers, respectively. Molecular docking indicated that the enhanced affinity of these compounds may be caused by the fact that this spacer length enables favorable interactions of the ligands with the orthosteric site of one monomer, and the allosteric site of the other monomer, as we reported previously for ropinirole-based bivalent ligands [21]. Compound **3a** is slightly too small to target both sites, whereas compounds **3d**–**3g** are too extended for an orthosteric–allosteric mode of interaction but still too small to target the orthosteric pockets of both monomers without crossing the membrane, which is theoretically possible but not favorable for steric reasons.

**Fig. 9:**
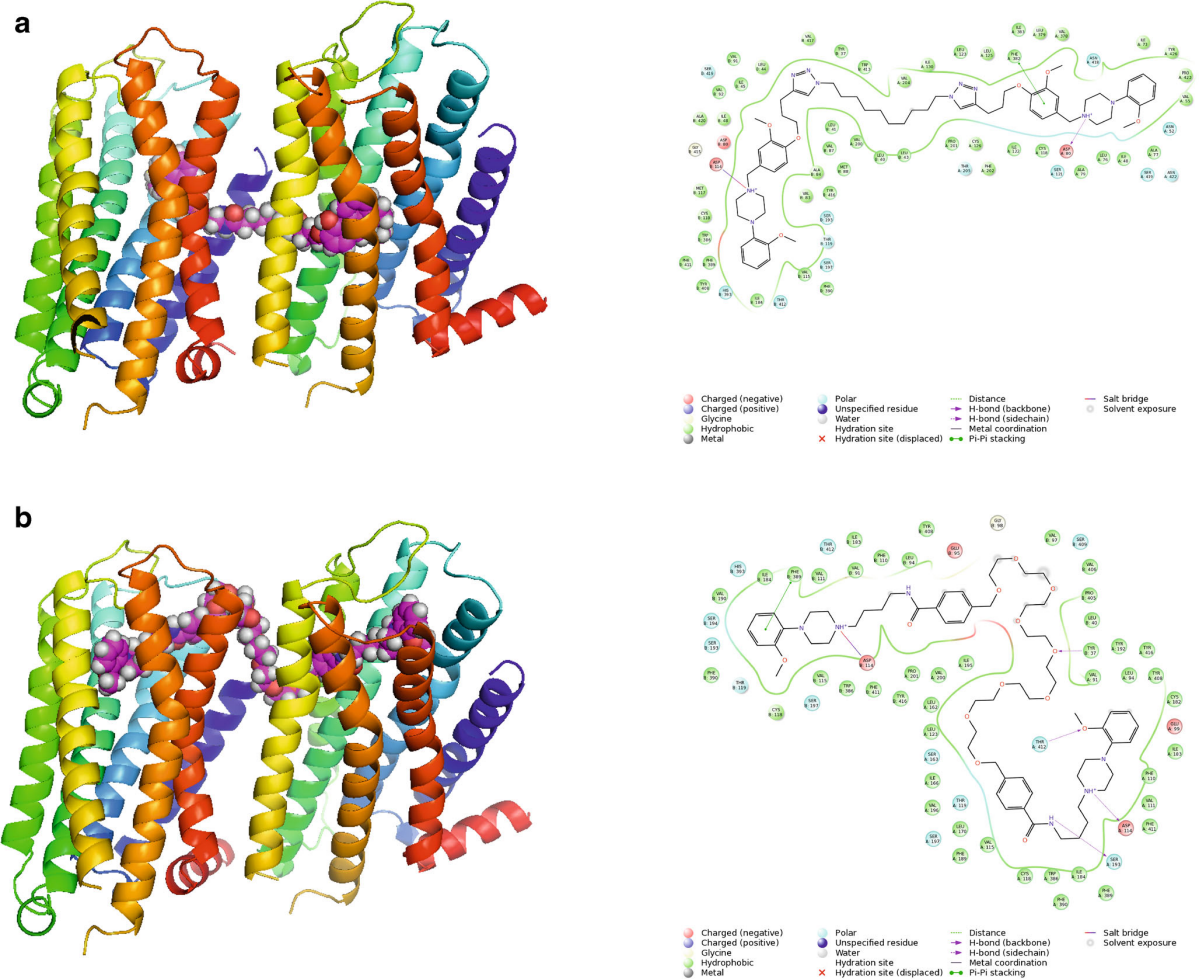
Different types of poses for bigger ligands. **a** Compound **4c** crosses the membrane to direct the other monomer (improbable pose). **b** Compound **5j** interacts with two orthosteric sites (a probable pose). Protein shown in* ribbon* representation, colored in spectrum mode. Ligands shown as* spheres* with* magenta carbon atoms*. 2D interaction maps also presented

The phenomenon that bivalent ligands occupy two orthosteric sites within the receptor dimer is controversial. Although bivalent ligands are designed to interact with both orthosteric sites, we demonstrated previously that ropinirole-based bivalent ligands are, in general, too small for such a binding mode and instead they may interact with the orthosteric site in one monomer and the allosteric site in the other monomer [21]. 1,4-Disubstituted aromatic piperidine/piperazine-based homobivalent ligands **4a–4i** were previously studied experimentally regarding their binding modes [23]. The 1,4-disubstituted aromatic piperazines **4a**–**4i** exhibited a change in the Hill slope in binding experiments for the homobivalent ligand compared to the monovalent ligands [23]. Gmeiner and co-workers postulated that a Hill slope of 2 indicates occupancy of both orthosteric sites of a D_2_R homodimer [23]. Based on the mathematical model, the steepening of the curve is the consequence of one bivalent ligand causing the displacement of two equivalents of the radioligand. It was claimed that these compounds are capable of interacting with two orthosteric sites, and that no allosteric modulation effect is connected with compound **4b**. However, the 1,4-disubstituted aromatic homobivalent piperazines **4a**-**4i** with the increased Hill slope did not display any improved affinity for the D_2_R compared to the monovalent counterparts, as would be predicted by the occupancy of two identical orthosteric ligands within a homodimer [23]. The interaction with two orthosteric sites is possible for these compounds only with the assumption that the compounds cross the membrane as in the case of compound **4c** (Fig. 9a). These results are in accordance with our earlier results for bivalent agonists of the D_2_ receptor [21]. The best potency of compounds **4e**, **4f** and **4i** can be attributed to a favorable linker length, enabling orthosteric–allosteric interactions within two receptor monomers.

In contrast, the clozapine homobivalent ligands **1a**–**1f**, **2a**–**2 g** and **3a**–**3 g**, showed no change in the Hill Slope, but a significant increase in binding affinity (75- and 79-fold) and functional potency (6- and 7-fold) [22]. In both cases, the docking of the homobivalent ligands into our D_2_R dimer model indicates that it is rather unlikely that both orthosteric sites can be engaged concurrently by one homobivalent ligand. The linker length of the synthesized ligands in both groups allows the homobivalent ligands only to interact at both orthosteric sites in a favourable binding mode when crossing the GPCR membrane. However, a binding mode including crossing of the membrane, although theoretically possible, is unlikely to occur in a biological system due to steric considerations. Therefore, our study provides further evidence that these ligands potentially show a bivalent (engaging of both orthosteric and allosteric binding sites) binding mode either within one protomer or across a homodimer. The change in the Hill slope to a steeper gradient that was observed for the 1,4-disubstituted aromatic piperazines, may be explained by positive cooperativity between receptor protomers within a receptor homodimer as our study indicates that is unlikely for these ligands to occupy both orthosteric sites simultaneously [21].

In case of compounds **5a**–**5n**, it was found that compounds **5a**–**5i** are capable of allosteric–orthosteric interactions whereas bigger compounds **5j**–**5n** are able to interact with both orthosteric sites (Fig. 9b). This is reflected by significantly better potency of compounds **5j**–**5n** in comparison to **5a**–**5i.**

### Molecular dynamics

**Fig. 10a–d:**
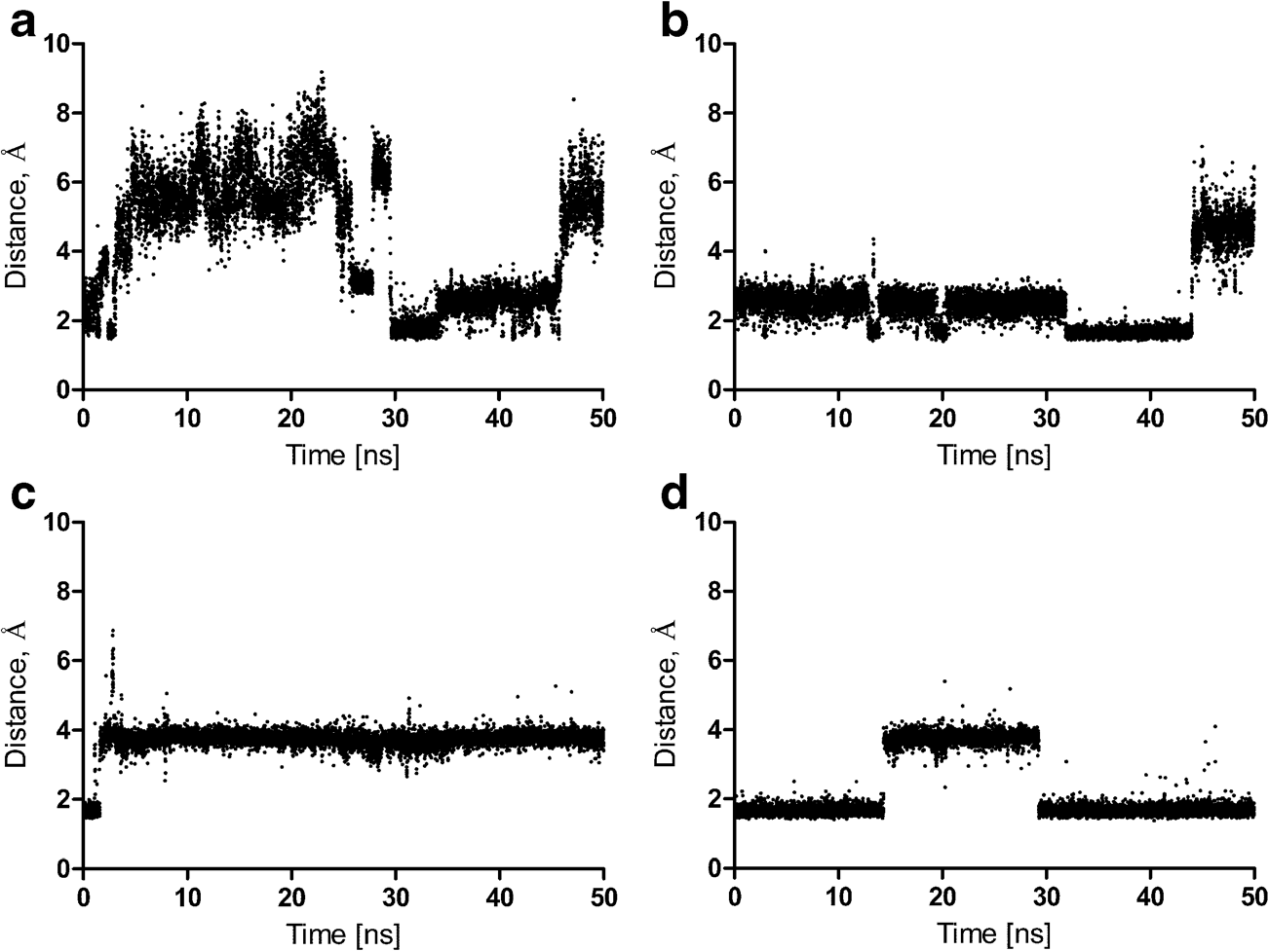
Changes between residues Arg 3.50 and Glu 6.30 forming ionic lock distances during 50-ns simulations. **a** D_2_ receptor homodimer apo form, monomer contributing TM4–TM5 to the interface. **b** D_2_ receptor homodimer in complex with **5j**, monomer contributing TM4–TM5 to the interface. **c** D_2_ receptor homodimer apo form, monomer contributing TM7–TM1 to the interface. **d** D_2_ receptor homodimer in complex with **5j**, monomer contributing TM7–TM1 to the interface

**Fig. 11a–f:**
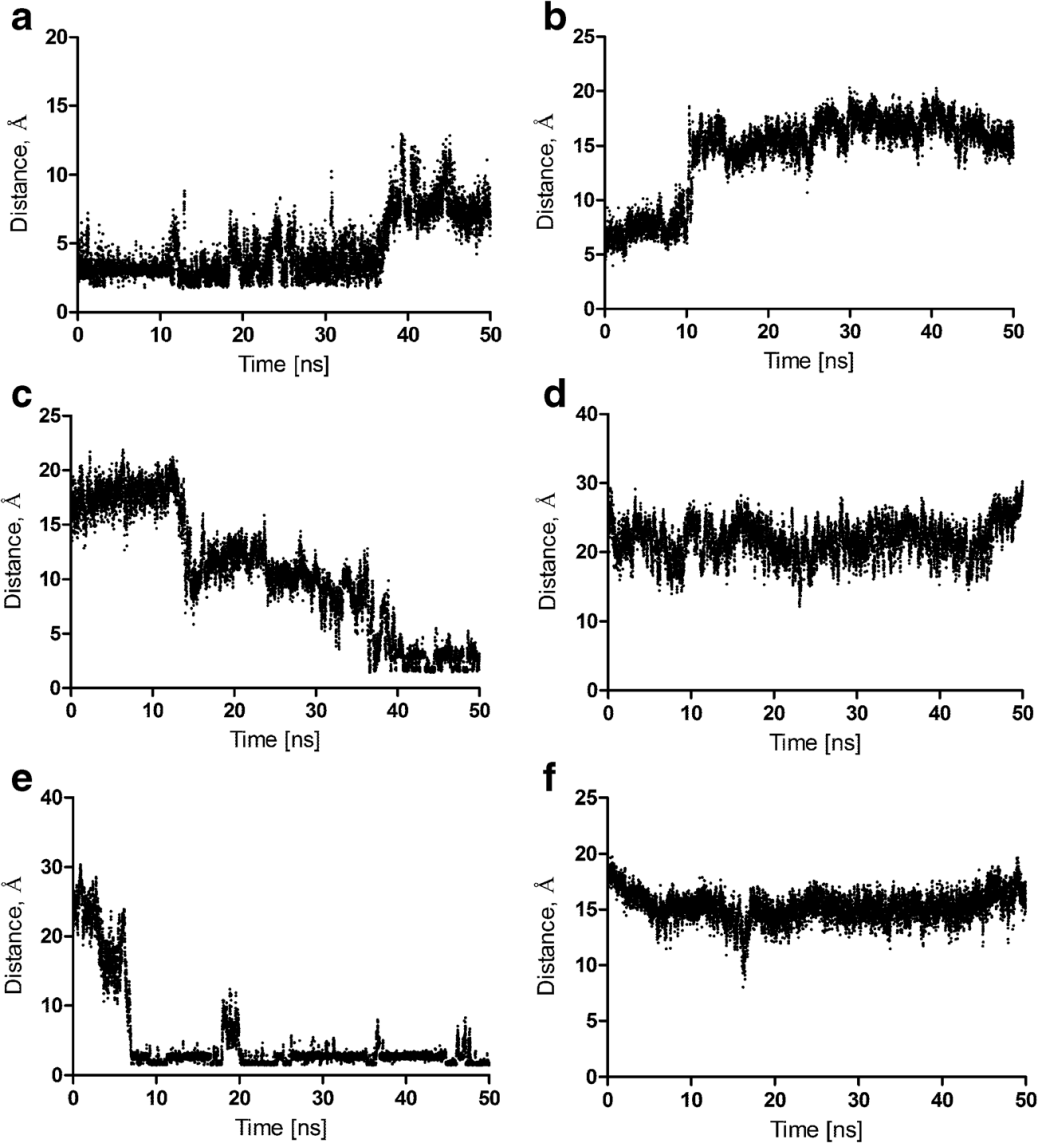
Destabilization of the homodimer interface by bivalent antagonist **5j**. **a**, **b** The distance between Tyr37 and Tyr 5.42 in the apo form (**a**) and in the complex with the ligand (**b**). **c**, **d** The distance between Arg145 and Cys443 in the apo form (**d**) and in the complex with the ligand (**d**). **e**, **f** The distance between Leu 5.65 and Lys 8.52 in the apo form (**e**) and in the complex with the ligand (**f**)

Molecular dynamics of all the ligand–receptor complexes in the POPC membrane was performed. The ligand–receptor complexes were in general stable (complex RMSD below 4 Å), and is presented for all the complexes in the Supporting Information. The effect of bivalent antagonists on the inactive receptor conformation was investigated (i.e., the change in distance between the ionic lock residues during 50 ns simulations). In the inactive state of family A GPCRs, there is a strong intramolecular interaction between residues Asp 3.49 and Arg 3.50 of the conserved (D/E)RY motif in TM3, and residues Glu 6.30 and Thr 6.34 in TM6 [53]. It was found that bivalent antagonists stabilize the receptor inactive conformation (Fig. 10). Moreover, it was determined that bivalent antagonists change the dopamine D_2_ receptor dimer interface by disrupting a set of hydrogen bonds that are weaker than in the apo form (Fig. 11). In particular, the following intersubunit hydrogen bonds are disrupted: Tyr37 (N terminus) – Tyr 5.42, Leu 5.65 – Lys 8.52, Arg145 (IL1) – Cys443 (C terminus), Fig. 11. However, the dimer interface is stabilized in a different way in the presence of bivalent ligands thanks to the increased number of water-mediated hydrogen bonds involving the ligands (in the extracellular and also intracellular part of the interface) in comparison to the dimer apo form.

**Fig. 12:**
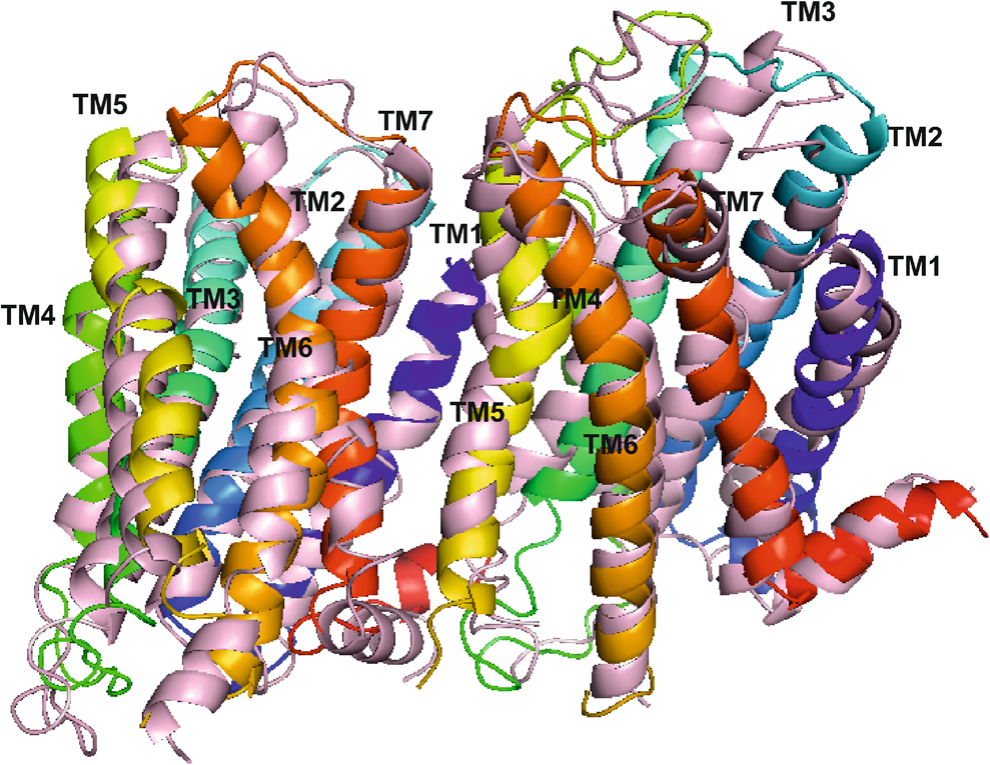
Superimposition of the D_2_ receptor homodimer apo form (protein in* ribbon* representation colored in spectrum mode, and D_2_ receptor homodimer in complex with 5j (protein in* ribbon* representation colored in* light pink*) after 50 ns molecular dynamics simulation. The RMSD of both structures is equal to 4.61 Å

In order to identify the changes in the D_2_ receptor homodimer conformation in the apo form and in complex with a bivalent ligand, the respective protein structures after 50 ns molecular dynamics simulations were superimposed (Fig. 12). The RMSD between the two structures was 4.61 Å. In the case of the monomer contributing TM7–TM1 to the interface (the left part of Fig. 12), it was observed that the part of TM5 close to the extracellular end approaches TM6 which in turn brings TM7 closer to the dimer in complex with the bivalent ligand compared to dimer apo form. Regarding the monomer that supplies TM4–TM5 to the interface, the greatest change was found for the TM3 part close to the extracellular end that points to TM2 in the dimer in complex with the bivalent ligand compared to the dimer apo form. The detailed analysis of the conformational changes in the dimer will be the subject of future studies. Concerning conformational changes of the bivalent ligands, in the case of a ligand, such as **5j**, interacting with both orthosteric sites, the ligand interacts stably with both Asp 3.32 and its linker, and its spacer parts possess a relatively greater conformational freedom, which contributes significantly to ligand RMSD (data not shown).

## Conclusions

In this work, we modeled the dopamine D_2_ receptor dimer in the inactive conformation using our previously elaborated multi-component protein–protein docking-based protocol. We found that the best scored dimer model has the TM4–TM5–TM7–TM1 interface, which is in agreement with experimental data [42, 43]. We used this model to study interactions of five sets of bivalent antagonists with the D_2_ receptor homodimer. We found that bivalent antagonists stabilize the receptor inactive conformation by maintaining the ionic lock interaction, and change the dimer interface by breaking a set of hydrogen bonds and maintaining another set of hydrogen bonds that are water- and ligand-mediated in the extracellular and intracellular part of the interface, respectively.

The docking study of ligands **1a–1f**, **2a–2 g**, **3a–3 g**, **4a–4i** and **5a–5n** into our dopamine D_2_ homodimer model revealed that most of the compounds tested are not able to interact at both orthosteric sites simultaneously. We determined a bitopic, orthosteric-allosteric type of interaction within one monomer for the smallest ligands, and the two monomers for medium-size ligands; only ligands **5j–5n** were able to interact in purely homobivalent conformation. Therefore, our work implies that it might be worth considering incorporating an allosteric pharmacophore into ligands with similar linker length (bitopic ligand) for the future design of ligands targeting the D_2_ receptor homodimer as well as other family A GPCRs homo- and heterodimers. In contrast, we determined the possibility that larger ligands may interaction with two orthosteric sites of a D_2_ homodimer; as a result, similar linker lengths should be considered for ligands with two orthosteric pharmacophores (homo- or heterobivalent ligands).

## Electronic supplementary material

Below is the link to the electronic supplementary material.

ESM 1 (DOCX 251 kb)
