# Supplementary material for: The dopamine D2 receptor dimer and its interaction with homobivalent antagonists: homology modeling, docking and molecular dynamics
Source: J Mol Model. 2016 Aug 4;22(9):203. doi: 10.1007/s00894-016-3065-2 (PMC5023759; doi:10.1007/s00894-016-3065-2)
Supplement: Supplementary file 1 — (DOCX 251 kb) [file 894_2016_3065_MOESM1_ESM.docx]

**The dopamine D_2_ receptor dimer and its interaction with homobivalent antagonists: homology modeling, docking and molecular dynamics**

Agnieszka A. Kaczor^1,2*^, Manuela Jörg^3^, Ben Capuano^3^

^1^Department of Synthesis and Chemical Technology of Pharmaceutical Substances with Computer Modeling Lab, Faculty of Pharmacy with Division for Medical Analytics, 4A Chodźki St., PL-20059 Lublin, Poland

^2^School of Pharmacy, University of Eastern Finland, Yliopistonranta 1, P.O. Box 1627, FI-70211 Kuopio, Finland

^3^Medicinal Chemistry, Monash Institute of Pharmaceutical Sciences, Monash University, 381 Royal Parade, Parkville, Victoria 3052, Australia.

E-mail: agnieszka.kaczor@umlub.pl

**Supplementary information**

Fig. S1 and S2: Scoring of dopamine D_2_ receptor dimer according to normalized parameter values and frequencies of best interfaces, respectively.

Fig. S3 – S9: RMSD of ligand-receptor complexes during 50 ns molecular dynamics.





**Fig. S1.** Scoring of dopamine D_2_ receptor dimer according to normalized parameter values. Scoring was performed for 28 interfaces (X axis) labeled according to transmembrane helices forming the interface, e.g. 12_12 for the interface formed by TM1 and TM2 from one monomer and TM1 and TM2 from the other monomer. A – Scoring according to Rosetta interface score; B – Scoring according to dimer interface area; C – Scoring according to free energy of binding; D – Scoring according to hydrogen bond energy.





**Fig. S2.** Scoring of dopamine D_2_ receptor dimer according to frequencies of best interfaces. Scoring was performed for 28 interfaces (X axis) labeled according to transmembrane helices forming the interface, e.g. 12_12 for the interface formed by TM1 and TM2 from one monomer and TM1 and TM2 from the other monomer. A – Scoring according to Rosetta interface score; B – Scoring according to dimer interface area; C – Scoring according to free energy of binding; D – Scoring according to hydrogen bond energy.





**Fig. S3.** RMSD of ligand-receptor complexes (measured using backbone atoms of the receptor and all atoms of the ligand) during 50 ns molecular dynamics for **1a** (A), **1b** (B), **1c** (C), **1d** (D), **1e** (E) and **1f** (F) and dopamine D_2_ receptor dimer.





**Fig. S4.** RMSD of ligand-receptor complexes (measured using backbone atoms of the receptor and all atoms of the ligand) during 50 ns molecular dynamics for **2a** (A), **2b** (B), **2c** (C), **2d** (D), **2e** (E) and **2f** (F) and the dopamine D_2_ receptor dimer.





**Fig. S5.** RMSD of ligand-receptor complexes (measured using backbone atoms of the receptor and all atoms of the ligand) during 50 ns molecular dynamics for **2g** (A), **3a** (B), **3b** (C), **3c** (D), **3d** (E) and **3e** (F) and the dopamine D_2_ receptor dimer.





**Fig. S6.** RMSD of ligand-receptor complexes (measured using backbone atoms of the receptor and all atoms of the ligand) during 50 ns molecular dynamics for **3f** (A), **3g** (B), **4a** (C), **4b** (D), **4c** (E) and **4d** (F) and the dopamine D_2_ receptor dimer.





**Fig. S7.** RMSD of ligand-receptor complexes (measured using backbone atoms of the receptor and all atoms of the ligand) during 50 ns molecular dynamics for **4e** (A), **4f** (B), **4g** (C), **4h** (D), **4i** (E) and **5a** (F) and the dopamine D_2_ receptor dimer.





**Fig. S8.** RMSD of ligand-receptor complexes (measured using backbone atoms of the receptor and all atoms of the ligand) during 50 ns molecular dynamics for **5b** (A), **5c** (B), **5d** (C), **5e** (D), **5f** (E) and **5g** (F) and the dopamine D_2_ receptor dimer.





**Fig. S9.** RMSD of ligand-receptor complexes (measured using backbone atoms of the receptor and all atoms of the ligand) during 50 ns molecular dynamics for **5h** (A), **5i** (B), **5j** (C), **5k** (D), **5l** (E), **5m** (F) and **5n** (G) and the dopamine D_2_ receptor dimer.
